# Inhalable CAR-T cell-derived exosomes as paclitaxel carriers for treating lung cancer

**DOI:** 10.1186/s12967-023-04206-3

**Published:** 2023-06-12

**Authors:** Wei Zheng, Tianchuan Zhu, Lantian Tang, Zhijian Li, Guanmin Jiang, Xi Huang

**Affiliations:** 1 Center for Infection and Immunity, Guangdong Provincial Key Laboratory of Biomedical Imaging, The Fifth Affiliated Hospital of Sun Yat-Sen University, Zhuhai, 519000 Guangdong China; 2 Department of Clinical Laboratory, The Fifth Affiliated Hospital of Sun Yat-Sen University, Zhuhai, 519000 Guangdong China; 3 Foshan Fourth People’s Hospital, Foshan, 528200 Guangdong China

**Keywords:** Lung cancer, CAR-T, Exosomes, Paclitaxel, Inhalation, Targeted delivery

## Abstract

**Background:**

Non-small cell lung cancer (NSCLC) is a worldwide health threat with high annual morbidity and mortality. Chemotherapeutic drugs such as paclitaxel (PTX) have been widely applied clinically. However, systemic toxicity due to the non-specific circulation of PTX often leads to multi-organ damage, including to the liver and kidney. Thus, it is necessary to develop a novel strategy to enhance the targeted antitumor effects of PTX.

**Methods:**

Here, we engineered exosomes derived from T cells expressing the chimeric antigen receptor (CAR-Exos), which targeted mesothelin (MSLN)-expressing Lewis lung cancer (MSLN-LLC) through the anti-MSLN single-chain variable fragment (scFv) of CAR-Exos. PTX was encapsulated into CAR-Exos (PTX@CAR-Exos) and administered via inhalation to an orthotopic lung cancer mouse model.

**Results:**

Inhaled PTX@CAR-Exos accumulated within the tumor area, reduced tumor size, and prolonged survival with little toxicity. In addition, PTX@CAR-Exos reprogrammed the tumor microenvironment and reversed the immunosuppression, which was attributed to infiltrating CD8^+^ T cells and elevated IFN-γ and TNF-α levels.

**Conclusions:**

Our study provides a nanovesicle-based delivery platform to promote the efficacy of chemotherapeutic drugs with fewer side effects. This novel strategy may ameliorate the present obstacles to the clinical treatment of lung cancer.

**Supplementary Information:**

The online version contains supplementary material available at 10.1186/s12967-023-04206-3.

## Background

Lung cancer is the most common cause of cancer-related deaths worldwide, with one of the highest mortality rates [1]. The major types of lung cancer include small-cell lung cancer (SCLC) and non-small cell lung cancer (NSCLC); the latter accounts for approximately 80–85% of all lung cancer cases [2].

Current lung cancer treatments include surgery, radiotherapy, and chemotherapy. Studies have provided evidence that 40–50% of the therapeutic response rate is achieved via chemotherapeutic treatment of NSCLC, among which paclitaxel (PTX) and platinum are the first-line strategies [3]. PTX inhibits cancer cell mitosis by inducing an increase in tubulin and triggering apoptosis. Although chemotherapeutic drugs such as PTX have achieved favorable potency in NSCLC, their further application is limited by various side effects including bone marrow hematopoietic system inhibition, liver and kidney toxicity, and severe gastrointestinal adverse reactions [4]. In addition, the clinical formulation of Taxol is a mixture of PTX and Cremophor EL (CrEL) that is dissolved in ethanol before intravenous injection [5]. Undesirably, this formulation and the route of administration often induces dose-dependent toxicity, injection extravasation and phlebitis [6]. Moreover, the nonspecific delivery of free PTX usually induces inflammation and enhances adverse reactions to the drug [7]. As a result, it is necessary to deliver PTX to the local tumor origin to improve therapeutic efficacy. Therefore, development of a novel therapeutic strategy is urgently needed to circumvent the weaknesses of the present administration mode and promote the targeted delivery of PTX.

Exosomes are small lipid bilayer nanoparticles secreted from various types of cells [8]. Because of their stable structure and unique mode of penetration, exosomes are considered an attractive drug delivery carrier that can be used to transport proteins, DNA, RNA, and drugs [9]. Notably, it has been reported that exosomes derived from T cells expressing chimeric antigen receptor (CAR-Exos) carried the CAR protein on their surface and contained granzyme B and perforin internally. Therefore, they can both recognize and kill tumor cells [10]. Clinical cases have proven the benefits of CAR-T therapy in various hematological tumors, which are attributed to the antigen-recognition of CAR and cytotoxic function of T cells [11–13]. In addition, CAR-Exos may have the potential to decrease the toxicity of PTX. First, CAR-Exos are only 30–150 nm in diameter and exhibit hydrophobic properties, meaning it can easily cross the physical barrier of the tumor to carry PTX into the tumor cells [14]. Second, engineered CAR on the surface can recognize tumor associated antigens, avoiding the cytotoxic effects of PTX on normal tissues. Finally, the targeted delivery of CAR-Exos may improve the bioavailability of PTX. Therefore, CAR-Exos may serve as a biocompatible nanoplatform that is expected to minimize systemic toxicity in the therapy of solid tumors.

Generally, intravenous administration is applied to deliver agents in the clinical treatment of lung cancer [15]. However, the biodistribution of exosomes introduced i.v. results in accumulation in metabolic organs such as the liver and spleen; very few reach the pulmonary tumor site [16, 17]. Compared with the conventional route, aerosol inhalation therapy has been widely adopted in various pulmonary diseases [18–23]. Many studies have shown that following inhalation, exosomes loaded with drugs mainly accumulated in the lungs, which may assist in drug release to the lungs and improved bioavailability [24, 25]. Therefore, inhalation is an attractive option for the targeted therapy for lung cancer as it may have better efficacy and less toxicity [26].

**Scheme 1.**
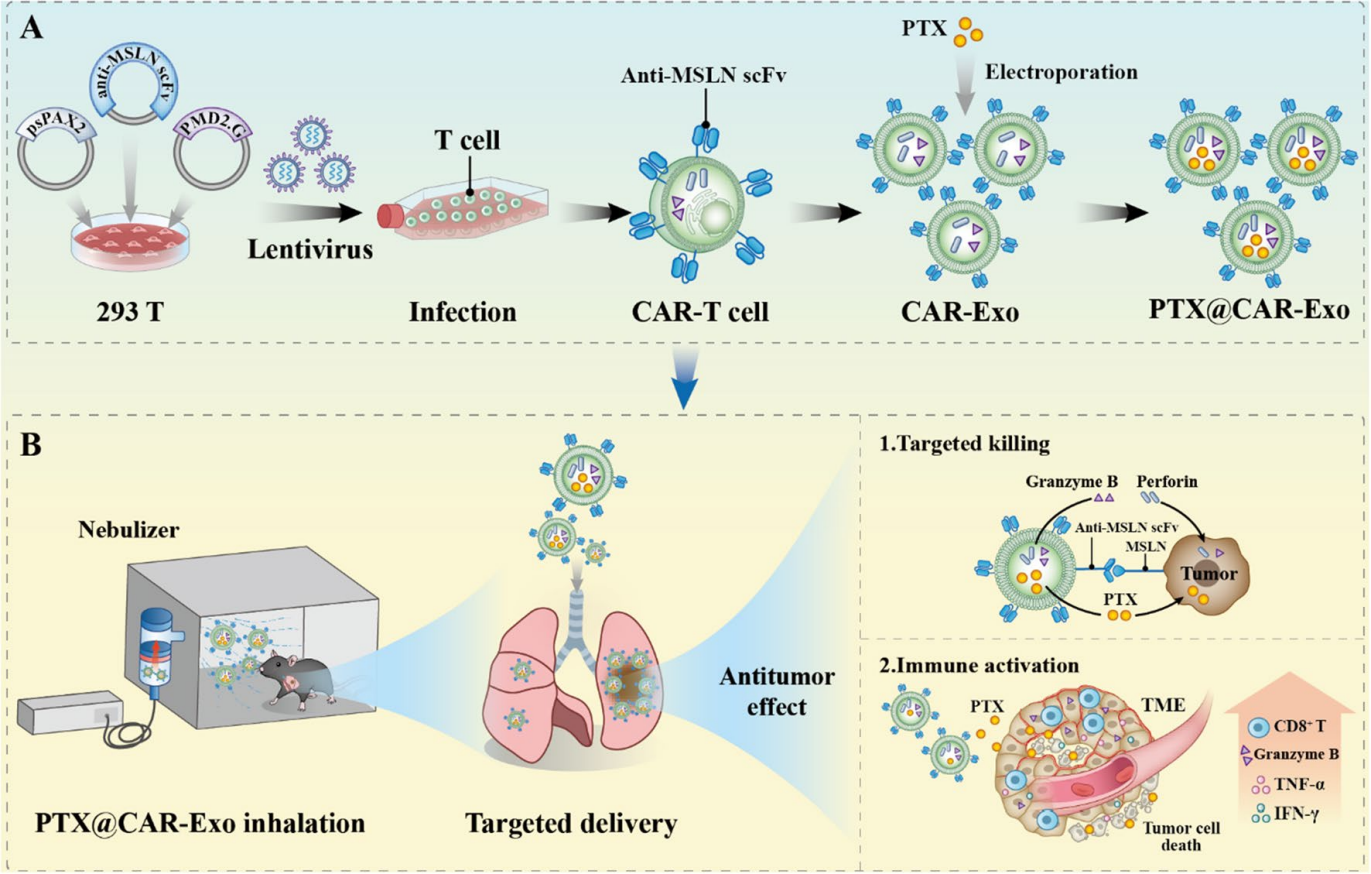
Schematic illustration of inhalable CAR exosomes derived from CAR-T cells as PTX carriers for treating lung cancer. **A** Preparation process of PTX@CAR-Exo. **B** Antitumor effect of inhalable PTX@CAR-Exo against lung cancer. CAR-T cell-derived exosomes (CAR-Exos) inherited the targeted cytolytic effects from parental cells by releasing granzyme B and perforins. The loaded PTX was delivered into the tumor area to stimulate immune responses. In contrast to conventional CAR-T cell therapy and intravenous administration of PTX, the inhalable PTX@CAR-Exos used in an orthotopic lung cancer model exhibited superior antitumor effects and improved the bioavailability of both CAR-Exos and PTX, with decreased systemic toxicity

Inspired by this, we proposed a novel therapeutic strategy to target orthotopic lung cancer via atomization inhalation of PTX-loaded CAR-Exos (PTX@CAR-Exos). As shown in the schematic diagram (Scheme 1), the prepared CAR-Exos were obtained from medium used to culture anti-mesothelin (anti-MSLN) expressing CAR-T cells. Then, the PTX was encapsulated in CAR-Exos to form PTX@CAR-Exos. In the C57BL/6J mouse orthotopic lung cancer model, a major percentage of inhaled PTX@CAR-Exos was observed in the lungs. Through the active recognition of CAR-Exos and the passive movement of inhalation, the treatment exhibited a better anti-tumor effect than free PTX, which was attributed to granzyme B and perforin from CAR-Exos and the therapeutic effects of PTX. Based on the above in vitro and in vivo results, we believe that chemotherapeutic drug-loaded CAR-Exos administrated via inhalation has great clinical potential for the treatment of NSCLC. This strategy may not only provide a nanocarrier that promotes the efficacy of anticancer drugs, but also reduce the present clinical adverse reactions of PTX chemotherapy in NSCLC.

## Methods

### Cells and plasmids

HEK 293 T and LLC cells (ATCC) were cultured in Dulbecco’s Modified Eagle’s Medium (DMEM, Thermo Fisher Scientific) containing 10% FBS (Gibco, USA) and 1% penicillin–streptomycin (Invitrogen) at 37 °C in a 5% CO_2_ environment. Stbl3 and DH5α competent cells were used for plasmid transformation.

The pCDH-CMV-MCS-EF1-GFP-Puro vector (cat. no. CD511B-1; System Biosciences, Mountain View, CA, US), psPAX2 (12260; Addgene), and pMD2.G (12259; Addgene) were used as the expression plasmid and the auxiliary plasmids for the lentivirus packaging system, respectively. These three plasmids were purchased from Shanghai Genery Biotechnology Company. The pCDH-CMV-MCS-Luc2-P2A-MEM-GFP-Puro plasmid was obtained by modifying pCDH-CMV-MCS-EF1-MEM-GFP-Puro in our laboratory, and was used to construct a stable lung cancer cell line expressing luciferase.

The nucleotide sequence encoding the MSLN protein was synthesized by BGI (Beijing, China). pCDH-MSLN-puro was obtained by cloning the MSLN sequence into the pCDH-CMV-MCS-Luc2-P2A-MEM-GFP-Puro vector. The target plasmid PCDH-MSLN-puro and two helper plasmids (psPAX2 and pMD2.G) were mixed at a 4:3:1 ratio. After 48 h, the lentivirus was collected and concentrated using polyethylene glycol 8000 (PEG 8000). Next, LLC cells were transduced with LV-MSLN-puro and cell lines were selected with puromycin to generate LLC cell lines stably expressing MSLN (MSLN-LLC). To assess MSLN expression, MSLN-LLC cells were incubated with biotinylated anti-MSLN antibody (Biolegend, San Diego, CA, USA) and then stained with PE-conjugated streptavidin (Biolegend, San Diego, CA, USA) for flow cytometric analysis.

### Vector construction and preparation of CAR-T cells

The anti-MSLN scFv nucleotide sequence [27] was derived from patent (No. US10640569B2), as published by Novartis. The gene sequence was synthesized by BGB (Beijing, China), and then a CD8α signal peptide was connected to the front end of anti-MSLN scFv. This connects the MYC tag, CD8 hinge, and transmembrane domains, a 4-1BB transactivation domain, and a CD3 zeta signaling domain. The MSLN-CAR was then obtained. The MSLN-CAR was inserted into the polyclonal site of pCDH-CMV-MCS-EF1-copGFP to obtain the corresponding lentiviral expression plasmid, pELNS-MSLN. Next, pELNS-MSLN was co-transfected into 293T cells with psPAX2 and pMD2.G at a 4:3:1 ratio. The lentivirus in the supernatant was collected 48 h later and then obtained by concentration with PEG 8000.

CD3^+^ T cells were collected by negative selection of peripheral blood PBMCs from healthy volunteers using a RosetteSep Kit (Stem Cell Technologies). All samples were collected according to the protocols approved by the review board of the Fifth Affiliated Hospital of Sun Yat-sen University, with written informed consent obtained from each donor. The sorted T cells were then cultured in X-VIVO-15 (Lonza) medium containing 10% fetal bovine serum and 100 U/mL recombinant human IL-2. Following stimulating with the T cell activator CD3/CD28 for 48 h, the above concentrated CAR lentivirus was added to infect T cells at a multiplicity of infection (MOI) of 10. After infection, the CAR-T cells were cultured in fresh medium (without FBS), and the supernatant was collected for the preparation of exosomes.

### Determination of the MOI of CAR-T cells

To determine the functional titer of lentiviruses containing a green fluorescent reporter (GFP) and CAR protein gene, 293T cells were transduced with serial dilutions of the concentrated lentiviral stock. The percentage of transduced cells was then assessed via flow cytometry at 72 h post-transduction [28]. The functional titer of the lentiviruses was calculated using the following equation: titer (TU/mL) = (F × C/V) × D, where F represents the percentage of GFP-positive cells, C denotes the cell number, V refers to the inoculum volume in milliliters, and D signifies the lentivirus dilution ratio. The optimal MOI for transducing T cells at the determined titer was subsequently established. An MOI gradient of 2.5, 5, 10, 20, and 40 was employed [29–32], and the corresponding viral stock solution volume was calculated as follows: MOI = titer (TU/mL) × lentivirus volume (mL)/cell number. Following T cell infection, GFP fluorescence was detected via flow cytometry, and the cell state was observed under a fluorescence microscope to identify the optimal MOI.

### Preparation of exosomes

Exosomes were harvested from culture supernatants through differential centrifugation. The cell culture medium underwent differential centrifugation (800×*g* for 5 min; 1200×*g* for 20 min; 10,000×*g* for 30 min) to remove cells and debris. The supernatant was collected, filtered using a 0.22-μm filter, and ultracentrifuged at 100,000×*g* for 60 min to pellet exosomes. The exosome particles were then washed with PBS and recovered through centrifugation at 100,000×*g* for 1 h. All centrifugation processes were performed at 4 °C, and samples were stored at − 80 °C until analysis. The purity of exosomes was assessed according to the particle/protein ratio (particle/mg) recommended by MISEV2018 [33]. Briefly, the protein concentration of exosomes (mg/mL) was determined using a BCA protein assay kit (Thermo Fisher Scientific), while the exosome concentration (particle/mL) was measured by nanoparticle tracking analysis (NTA).

### Cytotoxicity assay

The cytotoxicity of CAR-T cells and CAR-Exos toward target cells (MSLN-LLC) was assessed using the Lactate Dehydrogenase Assay (LDH assay) (Abcam, UK). Briefly, 1 × 10^4^ MSLN-LLC cells were seeded per well in 96-well plates. Following overnight incubation, CAR-T cells were incubated with MSLN-LLC cells at different E:T ratios. For CAR-Exos, different concentrations of exosomes were mixed with MSLN-LLC cells. After 24 h co-culture, the cell supernatant was measured for LDH reactivity by enzymatic reaction and measured with a microplate reader. Cell death was measured as a percentage of total LDH release, according to the manufacturer’s protocol.

### Live/dead assay with Calcein-AM/PI staining

The killing efficacy of PTX@CAR-Exo against MSLN-LLC cells was assessed using the Calcein-AM/PI Double Stain Kit (Sigma-Aldrich), which stains live and dead cells. Briefly, 1 × 10^5^ MSLN-LLC cells per well were seeded in 12-well plates. Then, 100 μL of PBS, 100 μg of T-Exos, 100 μg of CAR-Exos, 100 μL of PBS containing 10 μg of PTX, 100 μg of T-Exos containing 10 μg of PTX, and 100 μg of CAR-Exos containing 10 μg of PTX were added to the Petri dishes. After incubation for 24 h, the live and dead cells stained with Calcein-AM/PI were observed under an inverted fluorescence microscope (Olympus). Then, quantification was performed using ImageJ software.

### Exosome characteristics

The morphology and size of the CAR-Exo and PTX@CAR-Exo were examined by transmission electron microscopy (TEM, Tecnai G2 Sphera FEI 200 kV). Briefly, 10 μL of exosome was loaded onto a 300-mesh copper mesh, followed by negative staining using 2% aqueous uranyl acetate for 2 min at room temperature; excess solution was removed by suctioning with filter paper. After air-drying, the samples were imaged using transmission electron microscopy.

The size distribution and concentration of exosomes were analyzed by tracking Brownian motion using a NanoSight NS300 system (NanoSight NS300, Malvern, UK). The exosomes were diluted with PBS and added to the NanoSight sample chamber. The samples were continuously measured through the top plate of the flow cell at a steady speed, and the Brownian motion of nanoparticles was recorded three times (120 s each). The measured data were analyzed with the use of NTA 3.0 analysis software (Malvern).

The surface zeta potential of exosomes was measured using dynamic light scattering (DLS, ZEN 3600 Zetasizer, Malvern). The experiment was carried out according to the instructions in the manual.

### Flow cytometric analysis of exosomes

Exosomes were conjugated to aldehyde/sulfate latex beads with a diameter of 4 μm (catalog no. A37304, Invitrogen) in PBS overnight at 4 °C [34]. The mixture was then subjected to immunostaining and flow cytometric analysis [10]. The exosome-latex beads or cells were incubated with biotinylated protein L (Piscataway, NJ) to detect surface CAR expression. After incubation, staining was performed for 30 min using PE-conjugated streptavidin (Biolegend, San Diego, CA, USA) and the excess dyes were then washed. The kit (eBioscience) was used for fixation/permeabilization before intracellular staining for 45 min. The assay was carried out using an Attune NxT flow cytometer (Thermo Fisher Scientific) and data were analyzed using FlowJo software (version 10.8). The corresponding IgG antibody was used as an isotype control.

### Western blot analysis

Cells and exosomes were treated with lysis buffer (1% Triton X-100, 0.1% SDS, 0.1 M Tris HCl, pH 7, 1 mM PMSF, Sigma-Aldrich). Samples were then subjected to protein quantification using the BCA Protein Assay Kit (Thermo Fisher Scientific). Protein samples (20 μg/well) were loaded onto 10% SDS-PAGE gels and separated. Following transfer, the membranes were blocked with 5% skim milk or BSA for 1 h and washed twice with PBST, incubated overnight at 4 °C with the corresponding primary antibody, and then washed three times with PBST. The membranes were incubated with the corresponding horseradish peroxidase-coupled secondary antibodies (Cell Signaling Technology) for 2 h at room temperature. The immunoreactive bands were detected on a Mini-Medical/90 Developer (Image Works) using ECL chemiluminescence substrate (GE Healthcare). The following primary antibodies were applied: Calnexin (ab133615, Abcam), CD9 (ab263019, Abcam), CD63 (ab134045, Abcam), Hsp70 (ab181606, Abcam), TSG101 (ab125011, Abcam), GAPDH (390035, ZENBIO), Granzyme B (ab255598, Abcam), and Perforin (ab256453, Abcam).

### Laser confocal microscopy

To investigate the targeting of CAR-Exo to LLC cells overexpressing mesothelial (MSLN-LLC), CAR-Exo and MSLN-LLC were incubated together. Briefly, exosomes were stained with DiI (C1991S, Beyotime, Shanghai, China) at 37 °C for 25 min, purified by ultracentrifugation to remove free dye, and the labeled exosome weight was suspended in 100 μL of PBS after washing twice. Then, the exosomes were incubated with MSLN-LLC in a confocal dish for 4 h at 37 °C, washed three times with PBS, and the cells were fixed with 4% paraformaldehyde for 20 min. DAPI (C1002, Beyotime, Shanghai, China) was added to stain the nuclei of MSLN-LLC cells for 15 min. The cells were imaged under a confocal microscope (Zeiss LSM 880) to observe the fluorochromes with the following excitation (Ex) and emission (Em) wavelengths (GFP [Ex: 488 nm; Em: 530 nm], DiI [Ex: 549 nm; Em: 565 nm], DAPI [Ex: 364 nm; Em: 454 nm]).

### Preparation of PTX@CAR-Exos

PTX (MB1178, Dalian Meilun Biotech Co.) was loaded into the CAR-Exo using an electroporator (Scientz-2C, Ningbo SCIENTZ Biotech Co., Ltd., Ningbo, China) [35, 36]. Briefly, 1000 μg of exosomes and 1000 μg of PTX were mixed into 1 mL of PBS buffer containing 25 mM trehalose [37], then added to a chilled 4-mm electroporation cuvette and electroporated at 125 μF, 350 V, and 400 Ω. The cells were then incubated at 37 °C for 30 min to recover the exosome membranes. Next, paclitaxel-loaded exosomes were resuspended in PBS and dissociated at 120,000×*g* for 60 min at 4 °C to remove excess drug. Finally, the intensity of the specific paclitaxel absorption peak at 227 nm was measured by ultraviolet spectrophotometer, and the mass of paclitaxel loaded in exosomes was evaluated. The release efficiency of paclitaxel was calculated using the following the equation: Percentage of drug release (%) = OD value of the PTX released from the exosomes/OD value of the total PTX in the exosomes × 100%.

### Biocompatibility of exosomes in vivo

The animal experiments were approved and carried out in accordance with protocols approved by the Animal Care and Use Occasion of the Fifth Affiliated Hospital of Sun Yat-sen University. Healthy, C57BL/6J mice (5–6 weeks old) were obtained from the Guangdong Medical Experimental Animal Center.

The C57BL/6J mice were divided into four groups based on inhaled agents, which were PBS, 293-Exo, CAR-Exo, and PTX@CAR-Exo. All of the agents were administered at the same dose of 20 mg/kg using an aerosol inhalation instrument (YLS-8B, Yiyan Technology) on the 1st, 5th, and 10th days. The mice were sacrificed on day 14 post inhalation and major organs (lung, liver, spleen, heart, and kidney) were fixed in 4% paraformaldehyde buffer solution for H&E staining and histological analysis.

### Biodistribution of CAR-Exos

The CAR-Exos were incubated with the DiR (M5122, Abmole, USA) at 37 °C for 30 min, ultracentrifuged at 100,000×*g* for 60 min to remove free DiR, and DiR fluorescent dye-labeled exosomes were obtained. The exosomes were diluted in PBS and administered by aerosol through a mouse nebulizer. The mice were treated with different concentrations of CAR-Exos (5 mg/kg, 10 mg/kg, 15 mg/kg, 20 mg/kg, and 25 mg/kg) by aerosol inhalation, and then sacrificed 12 h after administration. To test the biodistribution of CAR-Exos at different time points, C57BL/6J mice were nebulized with CAR-Exos (10 mg/kg) and then sacrificed at each time point (1 h, 6 h, 12 h, 24 h, and 48 h) post administration. The Xenogen IVIS Spectrum system (Caliper Life Sciences, Hopkinton, MA) was used to detect the biodistribution of CAR-Exos in different organs (heart, lungs, liver, spleen, stomach, intestine, and kidneys) of the mice.

### Orthotopic lung cancer model with nebulized exosomes

For the orthotopic lung cancer model, MSLN-LLC cells suspended in Matrigel were injected into the left lungs of mice [38–40]. On the 7th day post injection, the mice were randomly divided into four groups: PBS, 293-Exo, CAR-Exo, and PTX@CAR-Exo. The mice inhaled different aerosol formulations through the mouse atomization instrument for 2 weeks. Lung tumor growth was measured on days 14 and 21. Mice were anesthetized with isoflurane and injected intraperitoneally with 100 mg/kg of luciferin solution (Abmole, USA). After 10 min, the Xenogen IVIS Spectrum system (Caliper Life Sciences, Hopkinton, MA) was used to detect the bioluminescent intensity of mouse lung tumors to determine tumor growth.

### Analysis of antitumor effect

Flow cytometry and ELISA assay analysis were performed to further verify the antitumor effects of the inhalation therapy. On day 14 of treatment, mice were sacrificed and tumors were collected.

To analyze the tumor immune cells, lung tissue was cut into pieces and digested with type IV collagenase (1 mg/mL, Sigma-Aldrich) at 37 °C for 60 min. The cell suspension was then filtered through a 100-mesh pore-size nylon mesh to remove insufficiently digested tissue. Cells were stained with the corresponding fluorescent antibodies for flow cytometric analysis. The antibodies used included: anti-CD45-PE/Cyanine7 (Biolegend, Clone: 30-F11), anti-CD3-FITC (Biolegend, Clone: 17A2), anti-CD4-APC (Biolegend, Clone: GK1.5), anti-CD8a-Brilliant Violet 421 (Biolegend, Clone: 53-6.7), anti-Granzyme B-PE (Biolegend, Clone: M1/70). Then, CD4^+^ T cells (CD45^+^CD3^+^CD4^+^), CD8^+^ T cells (CD45^+^CD3^+^CD8^+^), and CD8^+^GzmB^+^ T cells (CD45^+^CD3^+^CD8^+^GzmB^+^). The corresponding IgG antibody was used as an isotype control.

To analyze tumor cytokines, tissue was weighed according to the test indicators and instructions, ground in PBS (containing 1% PMSF), and then sonicated. After centrifugation at 12,000 rpm for 10 min, the supernatant was removed and analyzed via ELISA. To analyze the cytokines in peripheral blood, eyeball blood samples were collected in centrifuge tubes and centrifuged at 1000×*g* for 10 min after 30 min of agglutination. Then, the serum layers were removed and used for ELISA analysis. The below kits were used for cytokine evaluation: Mouse IL-6 ELISA Kit (EK206/3-96, Multi Sciences), Mouse IL-2 ELISA Kit (EK202/2-96, Multi Sciences), Mouse IFN-γ ELISA Kit (EK280/3-96, Multi Sciences), and Mouse TNF-α ELISA Kit (EK282/4-96, Multi Sciences).

### Statistical analysis

All experiments were conducted with at least three independent biological replicates, and data are presented as mean ± SD or mean ± SEM. Data analyses were performed using GraphPad Prism 8.0 Software (San Diego, CA). Two-tailed Student’s *t*-tests were used to compare two groups, whereas one-way analysis of variance followed by Tukey’s post-test was used for comparing more than two groups. The statistical significance was set as (*P < 0.05, **P < 0.01, ***P < 0.001, ****P < 0.0001, ns: not significant).

## Results

### Preparation and characterization of CAR-T cells

**Fig. 1:**
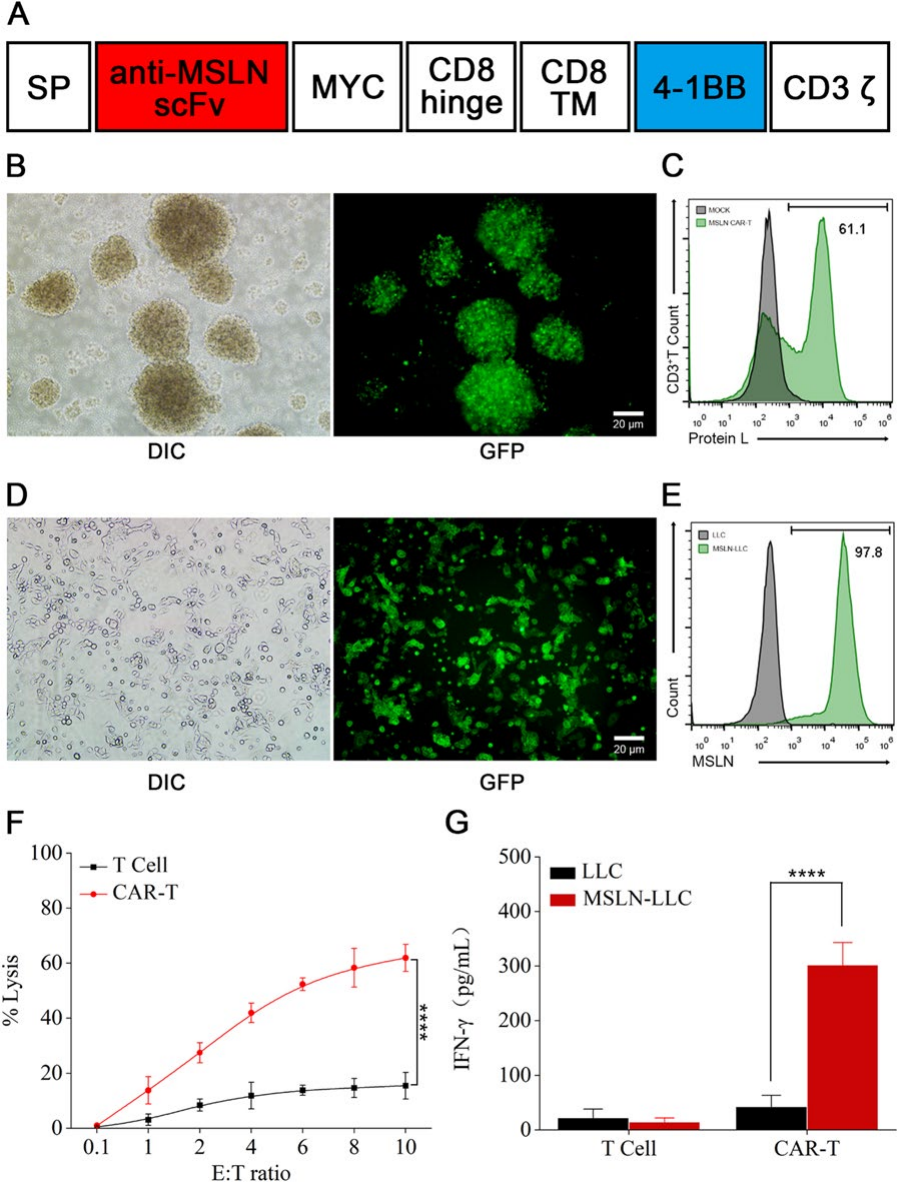
Preparation and characterization of CAR-T cells. **A** Molecular design of anti-MSLN mCAR. **B** The transfection efficiency of lentiviral in T cells was assessed by GFP expression using fluorescence microscopy. Scale bar: 20 μm. **C** Anti-MSLN CAR expression was assessed by Protein L staining and flow cytometry. Representative Protein L staining results are shown for mock transduced T cells and MSLN CAR-T cells. **D** Fluorescence images of LLC cells stably expressing GFP-MSLN after puromycin selection. Scale bar: 20 μm. **E** Representative flow cytometry histogram of the expression level of MSLN on LLC cells. An anti-MSLN antibody was used to confirm MSLN expression. **F** Cytolysis of CAR-T cells against MSLN-LLC cells. The cytotoxic activity of CAR-T and control T cells against cancer cell lines was assessed by an LDH-release assay at the indicated effector-to-target (E:T) ratios. **G** The level of IFN-γ evaluated by ELISA after co-culture of CAR-T cells with MSLN-LLC cells for 24 h. Data are presented as the mean ± SD of three biological replicates. *P < 0.05, **P < 0.01, ***P < 0.001, ****P < 0.0001, ns: not significant

To prove our hypothesis, we designed CAR-T cells expressing single-chain variable fragment (scFv) derived from antibodies that recognize mesothelin (MSLN). As shown in Fig. 1A, the second generation of anti-MSLN CAR also included a CD8α hinge and transmembrane domain. Both the 4-1BB and CD3ζ domains were in the intracellular region [41]. Next, the lentiviral vectors were constructed by cloning the CAR sequences into the backbone plasmid (pELNS) which contained the reporter gene (GFP) and MYC label. Then, the vectors were transfected into HEK293 to produce the lentivirus, followed by lentiviral infection with T cells. The high transduction efficiency was assessed using GFP expression under an inverted fluorescence microscope (Fig. 1B) and flow cytometric analysis using protein L confirmed the CAR expression on the surface of T cells with an efficiency of approximately 66% (Fig. 1C).

MSLN is highly expressed in multiple malignant cancers, including lung cancer [42, 43]. Thus, the stable MSLN-expressing LLC cell line (MSLN-LLC) was engineered through lentiviral transduction and puromycin selection to investigate the antitumor potential of anti-MSLN CAR-T. Following selection, green fluorescence seen under a fluorescence microscope suggested high expression of MSLN in LLC cells (Fig. 1D), which was in agreement with the flow cytometric analysis performed using an anti-MSLN antibody (Fig. 1E). The CAR-T cells were then cultured with MSLN-LLC cells at different E:T ratios and cytolysis was analyzed using a lactate dehydrogenase (LDH) detection kit. Compared to T cells, CAR-T cells exhibited a robust cytolytic effect in a dose-dependent manner in vitro (Fig. 1F). Further, increasing levels of IFN-γ were observed in CAR-T treated MSLN-LLC cells, but little change was observed for the same cytokine following treatment with normal T cells (Fig. 1G). These results revealed the specific tumor-targeting ability of the prepared anti-MSLN CAR-T cells in vitro.

### Preparation and characterization of CAR-Exos

**Fig. 2:**
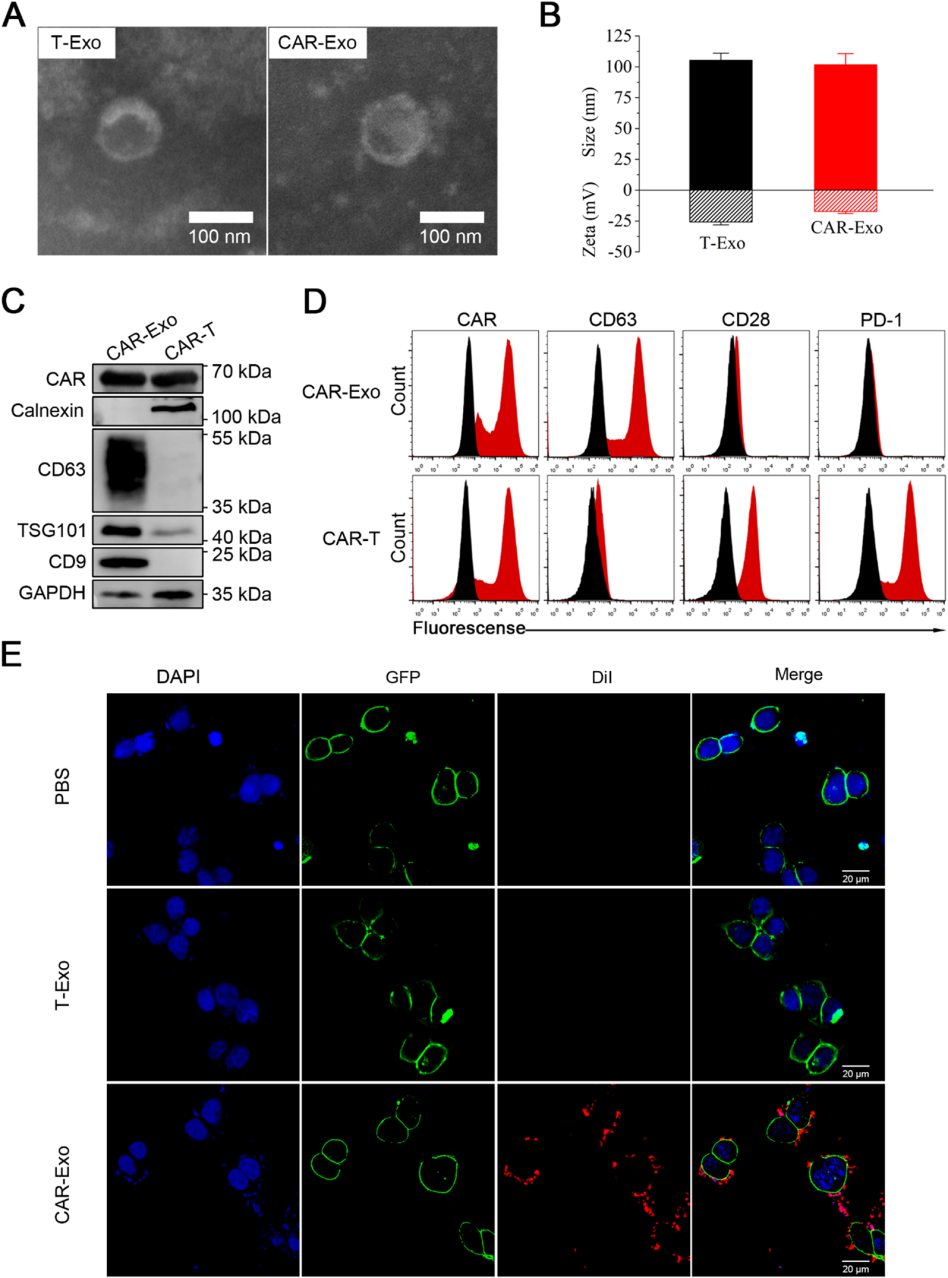
Preparation and characterization of CAR-Exos. **A** Representative TEM image of T-Exo and CAR-Exo. Scale bar: 100 nm. **B** Size distribution and zeta potential of T-Exos and CAR-Exos measured by NTA and DLS. Data are presented as the mean ± SD of three biological replicates. **C** The expression of CAR protein and exosomal markers in CAR-Exos or whole-cell lysates of CAR-T cells were analyzed by Western blotting. **D** Flow cytometric analyses of CAR-Exos conjugated to latex beads or CAR-T cells. The histograms shown isotype controls (black) and positive expression (red). **E** Representative Confocal laser scanning microscopy (CLSM) images of CAR-Exos bound to the surface of MSLN-LLC cells (green). T-Exos and CAR-Exos were labeled with DiI (red) and DAPI (blue) labeled nuclei of MSLN-LLC cells. CAR-Exos showed a higher binding affinity to the MSLN protein than T-Exos. Scale bar: 10 μm

After preparing CAR-T cells, exosomes were harvested from the supernatant using differential ultracentrifugation (UC). MISEV2018 guidelines recommend particle/protein, particle/lipid, and lipid/protein as purity measures; however, most practical studies utilize particle/protein as a purity indicator. The results (Additional file 1: Fig. S1) demonstrated that the purity of different CAR-Exo batches was always greater than 10^11^ particles/mg, which aligns with the exosome purity level required for preclinical studies [44, 45]. Additionally, transmission electron microscopy (TEM) and nanoparticle tracking analysis (NTA) measurements confirmed that the prepared exosomes were homogeneous, with a round or oval morphology, and their peak size was approximately 100 nm, which is consistent with the typical characteristics of exosomes (Fig. 2A, B) [8]. The zeta potential of CAR-Exos was − 17.3 mV as shown by dynamic light scattering (DLS) compared to the − 25.7 mV from T-Exos (Fig. 2B), and the zeta potential of exosomes from CAR-T cells was marginally higher than those from T cells, indirectly indicating successful CAR protein expression on the exosomes. The protein characterization of CAR-Exos, including the assessment of exosomal markers, was performed using western blotting (Fig. 2C). According to the International Society for Extracellular Vesicles (MISEV2018) guidelines, the general characterization of EVs should include at least three positive protein markers of EVs, comprising at least one transmembrane/lipid-bound protein (e.g., CD63 or CD9) and one cytosolic protein (e.g., TSG-101), as well as at least one negative protein marker (e.g., Calnexin) [33]. In our study, we observed the presence of exosome biomarkers CD9, TSG-101, and CD63, while the endoplasmic reticulum biomarker Calnexin was not detected in CAR-Exos. This observation underscores the quality of the exosomes and the absence of endoplasmic reticulum contamination. Additionally, the detection of CAR in the exosomes derived from parental cells highlights their potential for targeting MSLN-expressing tumors. In addition, to measure the direction of the binding domain, flow cytometry was performed, which proved that after coupling to the latex beads, the scFv on the surface of exosomes was oriented outward. Meanwhile, high levels of both CAR and CD63 were observed in CAR-Exos, where CD28 was expressed at relatively low levels and the PD-1 receptor was undetectable (Fig. 2D). These results were consistent with published reports [10].

Next, the CAR-Exos were cultured with MSLN-LLC cells to further investigate their targeting ability in vitro. The cell membrane was labeled with green fluorescence, as the engineered MSLN-LLC harbored a GFP label on the C terminus of MSLN. The exosomes were stained with DiI, and the nuclei of MSLN-LLC cells stained with DAPI to provide a purple fluorescent label. PBS was processed as the blank control. Confocal images showed that CAR-Exos effectively bound around the surface of MSLN-LLC cells, while no specific binding was observed in the control group treated with T-Exos (Fig. 2E). Together, these findings indicated that CAR-Exos may target tumor cells through the interaction between the anti-MSLN scFv on the surface and the MSLN on LLC cells.

### The enhanced antitumor activity of PTX@CAR-Exos in vitro

**Fig. 3:**
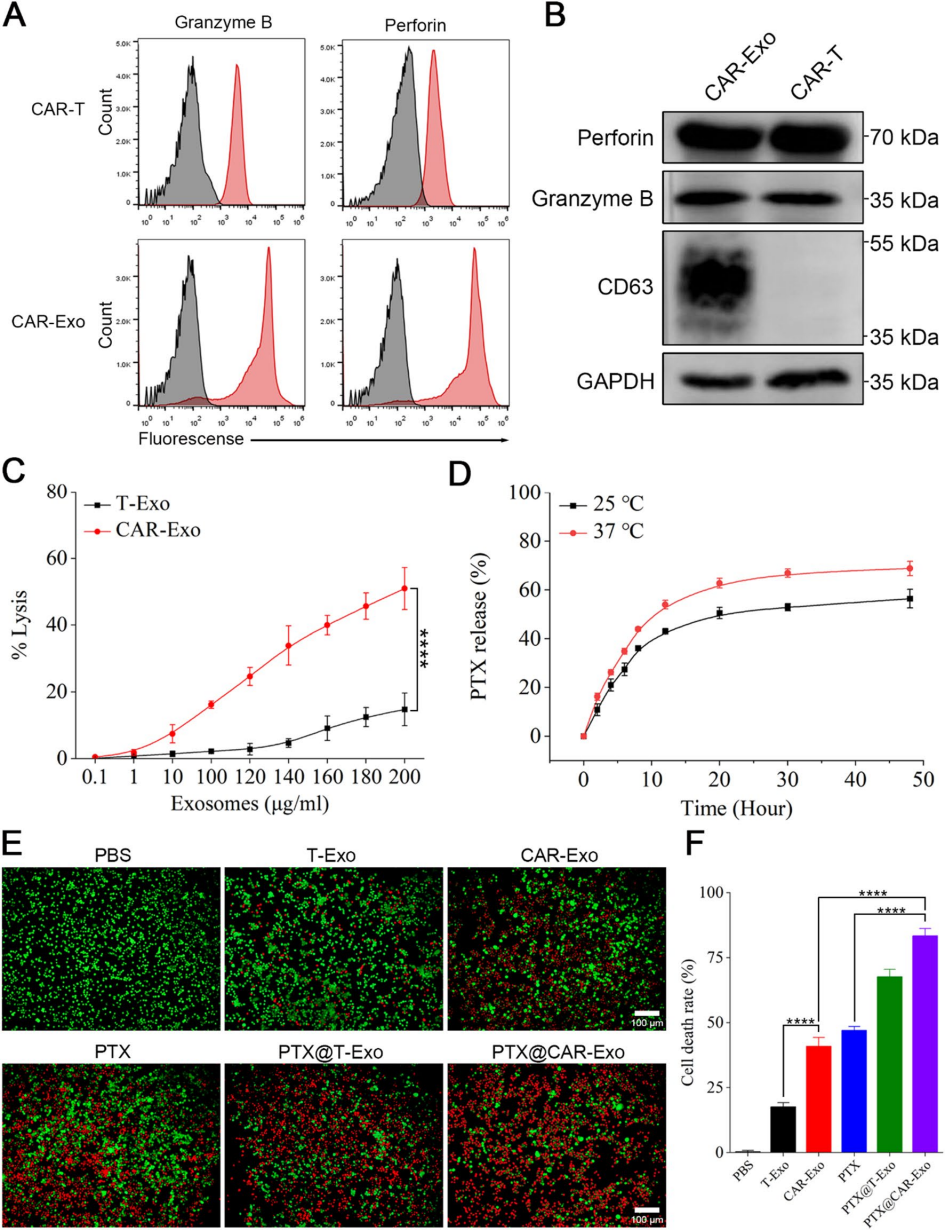
The enhanced antitumor effect of PTX@CAR-Exos in vitro. **A** Flow cytometric analyses of granzyme B and perforin expression of CAR exosomes conjugated to latex beads or CAR-T cells. The histograms shown isotype controls (black) and positive expression (red). **B** The expression of granzyme B and perforin in CAR-Exos or whole-cell lysates of CAR-T cells were analyzed by western blotting. **C** The cytotoxic activity of different concentrations of CAR exosomes against MSLN-LLC cells. **D** In vitro drug release profile of paclitaxel-loaded CAR exosomes was evaluated in PBS at pH 7.4 and different temperatures. The percentage of drug release (%) = OD value of the PTX released from the CAR exosomes /OD value of the total PTX in the CAR Exosomes × 100%. **E** Cytotoxicity and Calcein-AM/PI staining analysis in vivo. Fluorescent microscopic imaging of MSLN-LLC cells after treatment with PBS, T-Exos, CAR-Exos, PTX, PTX@T-Exos, or PTX@CAR-Exos for 24 h, cells were stained with Calcein-AM/PI (original magnification, 10 ×). Scale bar: 100 μm. **F** Quantitative analysis of cell death rate analyzed by Calcein-AM/PI staining. Data are presented as the mean ± SD of three biological replicates. *P < 0.05, **P < 0.01, ***P < 0.001, ****P < 0.0001, ns: not significant

Whether CAR-Exos inherited the anticancer effect of CAR-T required further investigation. As shown in Fig. 3C, CAR-Exos suppressed MSLN-LLC in the range of 0.1–200 μg/mL, as determined using an LDH kit. There was some evidence demonstrating that CAR-T cells exert their tumor-killing effects by producing granzyme B and perforin after recognition [46]. Thus, whether the anticancer activity was carried through these cytotoxic effectors required further study. The flow cytometric analysis showed that both granzyme B and perforin were expressed in CAR-T cells and their released CAR-Exos (Fig. 3A). This result was supported by western blotting (Fig. 3B), as described in previous studies [10].

Following confirmation of targeting effect and killing capacity of CAR-Exos, we loaded PTX into exosomes via electroporation to obtain PTX@CAR-Exos. Both TEM observation and NTA analysis showed that exosome characteristics were unaffected after drug encapsulation (Additional file 1: Fig. S2). The drug-loaded exosomes were still homogeneous, with a round or oval morphology, and their peak size was approximately 120 nm, which is consistent with the typical characteristics of exosomes. Moreover, the particle size and zeta potential of CAR-Exos experienced only a minor increase after paclitaxel loading, suggesting that the drug loading did not compromise the exosomes' stability. To analyze the drug (PTX) release efficiency of PTX@CAR-Exos, we identified a specific absorption peak at 227 nm of PTX using spectrophotometry and calculated the concentration of PTX based on the standard curve equation (Additional file 1: Fig. S3). As shown in Fig. 3D, drug release in vitro was evaluated in PBS at pH 7.4 and 37 °C. Results indicated that PTX in CAR-Exos was released at a rapid rate within the first several hours and up to 50% after 10 h. Then, the rate remained stable, suggesting that the PTX@CAR-Exos possessed a well-sustained release performance, as expected. This could prolong the retention of chemotherapeutic drugs. Moreover, PTX@CAR-Exos was stored at − 80 °C for 1 month with little particle size change, indicating its stability (Additional file 1: Fig. S4).

To further explore whether the chemotherapeutic efficacy of PTX loaded into CAR-Exos enhanced antitumor activity, MSLN-LLC cells were treated with PBS, T-Exos, CAR-Exos, PTX, PTX@T-Exos, and PTX@CAR-Exos. After 24 h, cell viability was monitored by staining with Calcein-AM (green, to identify live cells) and Propidium Iodide (PI, red, to identify dead cells). In contrast to PBS and T-Exos, high-intensity red fluorescence was observed in the CAR-Exos treated group, indicating significant cytolysis of MSLN-LLC cells. This may have been driven by the targeted recognition of CAR from the CAR-Exos. Notably, the PTX@CAR-Exos exhibited the best killing effect among the above-mentioned groups (Fig. 3E). This may be attributed to the combined effects of both PTX and CAR-Exos. In addition, the percentage of cytolysis was determined using ImageJ software. Results showed that the death rate of MSLN-LLC cells was significantly increased in the PTX@CAR-Exos treated group, reaching approximately 83.37% (Fig. 3F). All these data revealed that PTX@CAR-Exos exerted targeted delivery to improve the efficacy of chemotherapeutic drugs and enhance the elimination of tumor cells.

### The biodistribution of inhaled CAR-Exos in vivo

Although PTX@CAR-Exos exhibited pronounced antitumor activity in vitro, the in vivo therapeutic effect required further study. Initially, we evaluated the biocompatibility of inhaled PTX@CAR-Exos in vivo by examining potential organ damage in mice. The C57BL/6J mice were randomly divided into four groups and inhaled either PBS, T-Exos, CAR-Exos, or PTX@CAR-Exos. Then, the mice were sacrificed and their major organs were subjected to H&E staining and histological analysis. The results demonstrated no pathological changes in the primary organs (heart, liver, spleen, lung, and kidney) of mice in either the control group or the PTX@CAR-Exo group (Additional file 1: Fig. S5 A). Meanwhile, no significant abnormalities were detected in body weight (Additional file 1: Fig. S5 B) or biochemical measurements of liver injury (Additional file 1: Fig. S5 C–E) and kidney function (Additional file 1: Fig. S5 F, G) in mice, implying the superior biocompatibility of PTX@CAR-Exos in vivo.

**Fig. 4:**
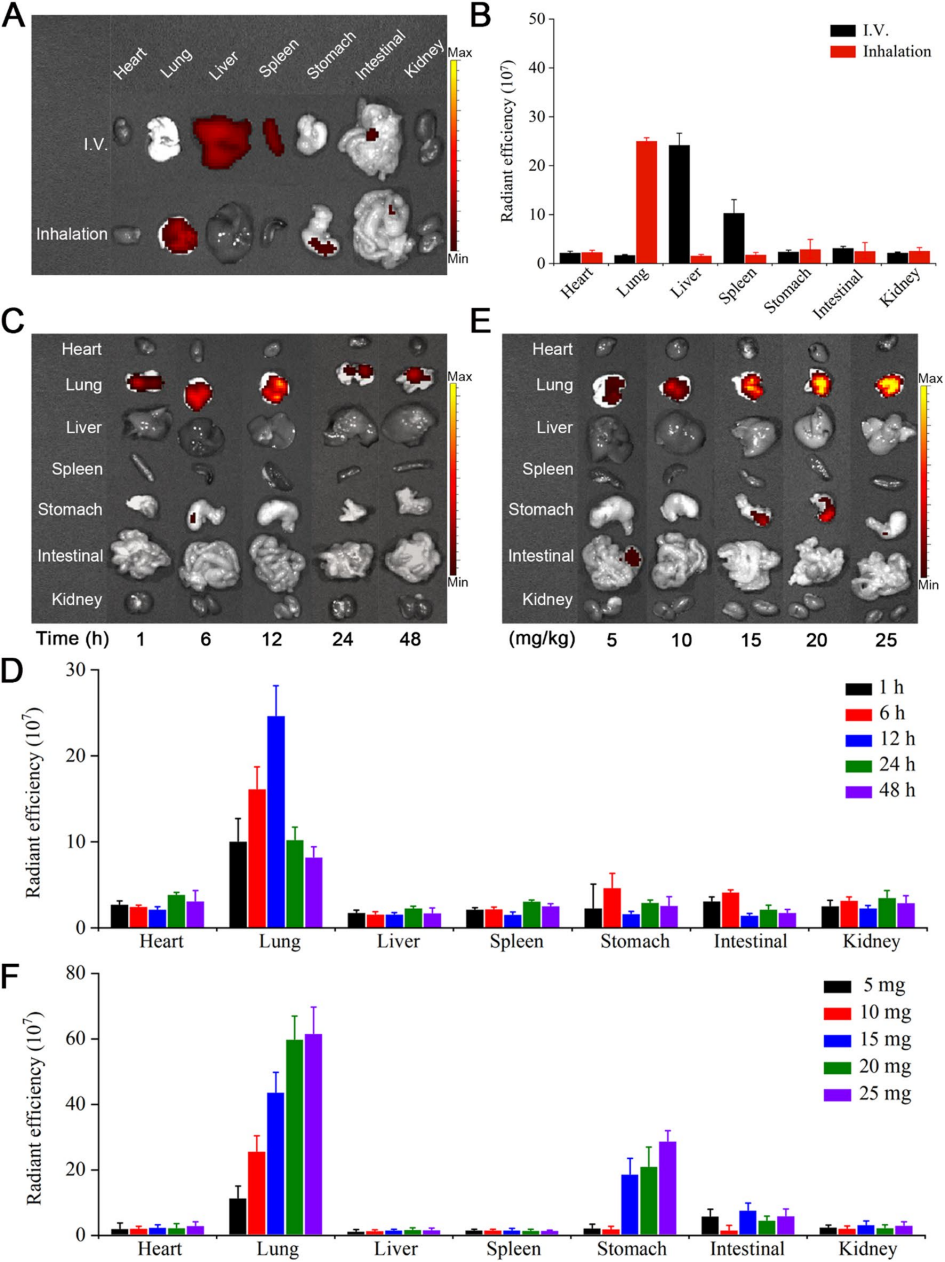
The biodistribution of DiR-labeled CAR-Exos via inhalation in vivo. **A** The biodistribution of CAR-Exos in vivo via intravenous injection or inhalation. **B** The quantification of DiR intensity from **A**. **C** The intensity of inhaled CAR-Exos at different time points in vivo. **D** The quantification of DiR intensity at different time points. **E** The intensity of inhaled CAR-Exos at different concentrations of exosomes in vivo. **F** The quantification of DiR intensity at different doses. Data are presented as the mean ± SD of three biological replicates

To explore the bioactivity of PTX@CAR-Exos in mice treated with inhaled therapeutics, biodistribution was monitored via different administration approaches. Almost all DiR-labeled CAR-Exos inhaled through a vibrating mesh nebulizer were enriched in the lungs, while the intravenous exosomes mainly accumulated in the liver and kidneys (Fig. 4A, B). The retention of CAR-Exos was further evaluated by detecting the fluorescence of stained exosomes at different time points. The images showed that the highest fluorescence was found at 12 h post inhalation and after 24 h, the signal intensity reduced gradually (Fig. 4C, D). Next, different concentrations of CAR-Exos were administered via inhalation, and the fluorescence of each organ was monitored at 12 h after administration. The results showed that in the range of 5–20 mg/kg of inhaled exosomes, the fluorescent intensity increased, followed by unchanged intensity between 20 mg/kg and 25 mg/kg (Fig. 4E, F). In addition, a slight fluorescence was detected in the stomach. This may be due to swallowing during inhalation, which allowed the exosomes to enter the gastrointestinal tract orally [19]. These data indicated that inhaled exosomes accumulated in the lungs in a time- and dose-dependent manner.

Furthermore, the orthotopic lung cancer model was established using MSLN-LLC cells to explore the biodistribution of inhalable CAR-Exos. The IVIS system showed that the DiR-labeled CAR-Exos were localized in the tumor area of the pulmonary site, as expected (Additional file 1: Fig. S6), indicating that they had targeting ability.

### The therapeutic efficacy of PTX@CAR-Exos in an orthotopic lung cancer model

**Fig. 5:**
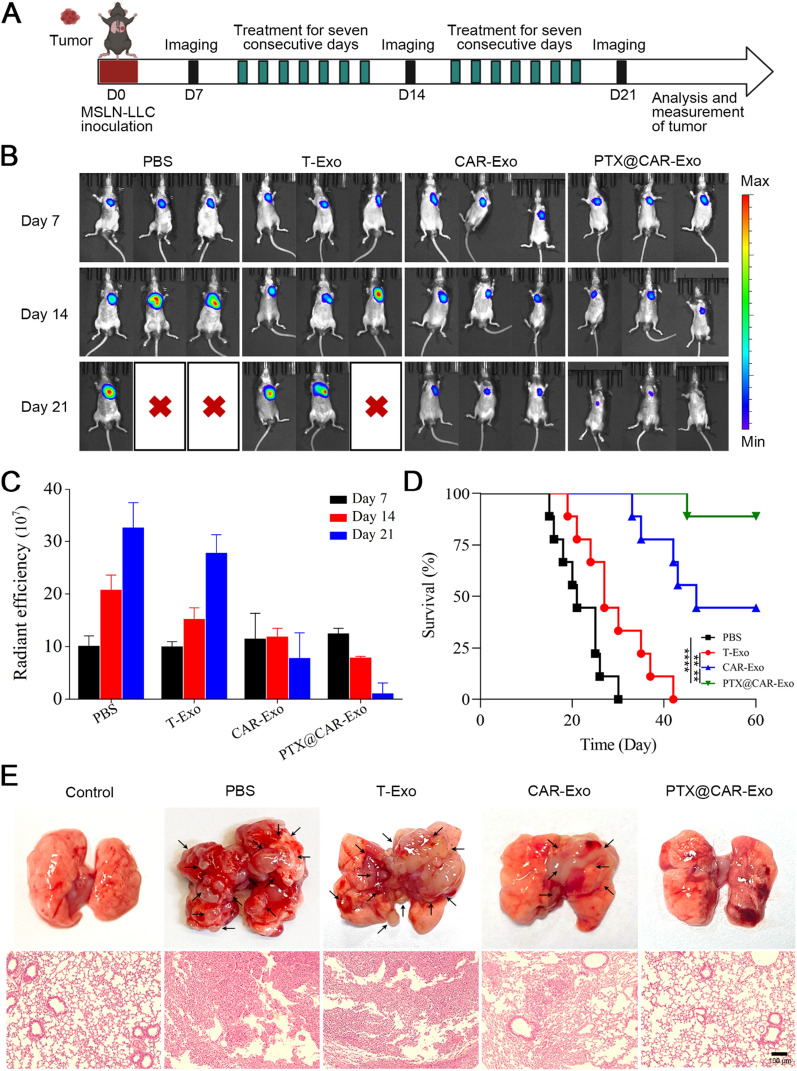
The therapeutic efficacy of PTX@CAR-Exos in orthotopic lung tumor-bearing mice. **A** The designed diagram for evaluating the efficacy of PBS, T-Exos, CAR-Exos, or PTX@CAR-Exos treated orthotopic lung tumor-bearing mice. Mice were given inhaled therapy via nebulizer for 2 weeks, starting on day 7 after establishment of the orthotopic model; IVIS imaging was performed on days 14 and 21 post implantation. **B** Respective bioluminescence images of MSLN-LLC cells in mice on days 7, 14, and 21 post implantation. **C** Statistical analysis of the signal intensity from **B**. **D** Survival analysis of tumor bearing mice after different treatments. **E** Representative images of tumor nodules in different groups and H&E-stained slice images of the lungs. Scale bar: 100 μm. Data are presented as the mean ± SD of three biological replicates. *P < 0.05, **P < 0.01, ***P < 0.001, ****P < 0.0001, ns: not significant

As described above, the antitumor effect was further explored following the establishment of an orthotopic lung cancer model, as shown in the diagram (Fig. 5A). The mice bearing MSLN-LLC cells were divided into four groups according to treatment: PBS, T-Exos, CAR-Exos, and PTX@CAR-Exos. The vibrating mesh atomizer was used for the inhaled treatment administering a dose of 20 mg of CAR-Exos per kilogram of mouse for two consecutive weeks (Additional file 1: Fig. S7). The biodistribution of MSLN-LLC cells in the mice was detected by IVIS system on day 14 or 21 post inhalation. As shown in Fig. 5B and  C, CAR-Exos inhibited tumor size with administration by inhalation. Compared with the CAR-Exos group, a much smaller tumor size was observed in the inhaled PTX@CAR-Exos treatment group, with no recurrence. Further, an extended survival rate was achieved in the PTX@CAR-Exos group compared with the CAR-Exos group (Fig. 5D). Mice treated with PBS or T-Exos exhibited a pulmonary structure that was significantly damaged by the density of the tumor nodules. However, an apparent decrease in tumor area was observed in the CAR-Exos treatment group. Notably, lung structure was recovered following PTX@CAR-Exos inhalation, accompanied by almost normal alveoli (Fig. 5E). Therefore, PTX@CAR-Exos possessed a significant, targeted cytotoxic effect on tumors in vivo.

### Reprogramming of the tumor microenvironment by PTX@CAR-Exos in vivo

**Fig. 6:**
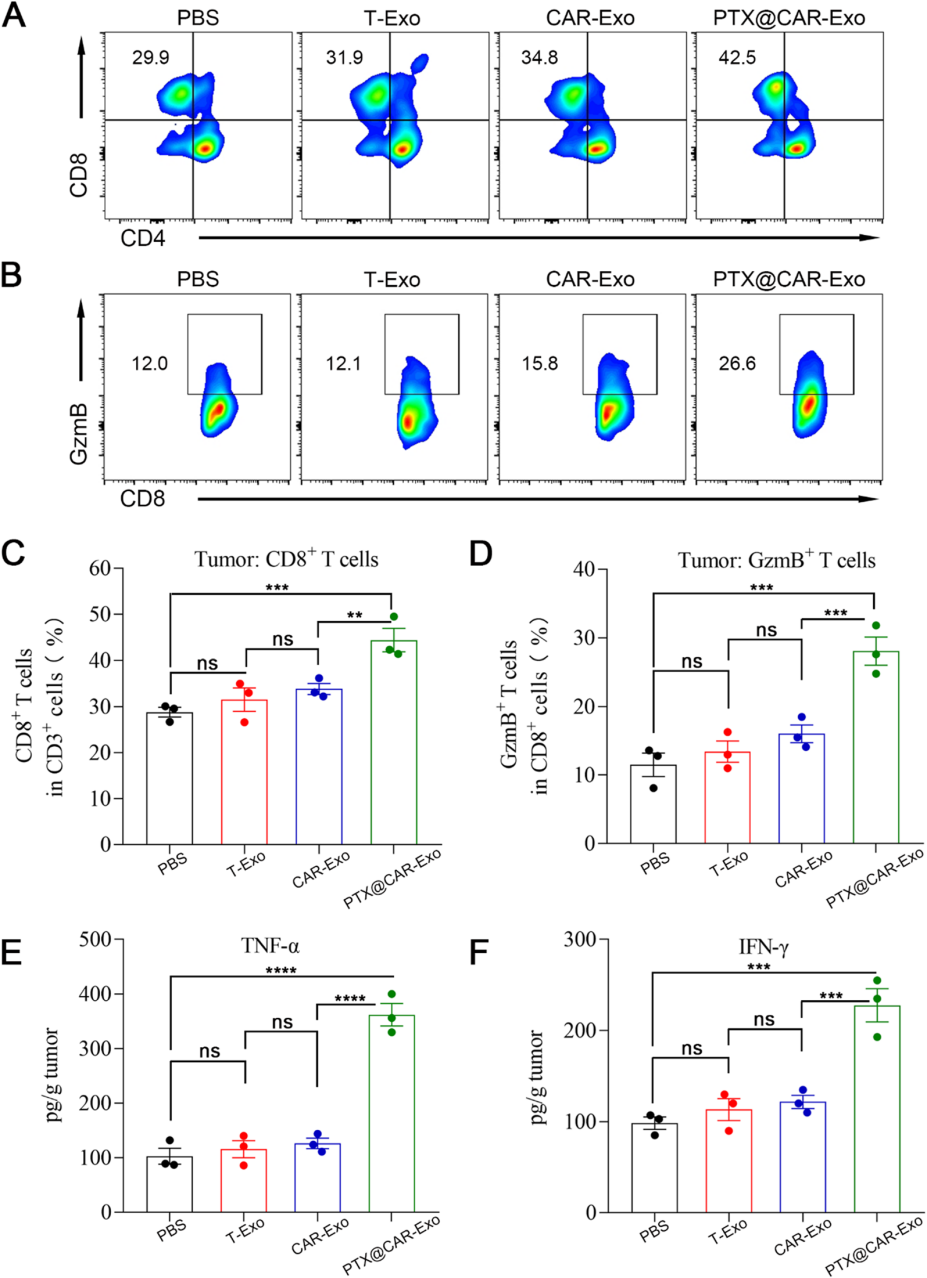
Improvement in the tumor microenvironment following PTX@CAR-Exos treatment in vivo. **A**, **C** Representative flow cytometry plots and statistical analysis of CD4^+^ T cells and CD8^+^ T cells infiltration at the tumor site. **B**, **D** Representative flow cytometry plots and statistical analysis of infiltrated CD8^+^GzmB^+^ T cells at the tumor site. Cytokine concentrations **E** TNF-α and **F** IFN-γ in tumor tissues as quantified by ELISA assay. Data are presented the mean ± SEM. *P < 0.05, **P < 0.01, ***P < 0.001, ****P < 0.0001, ns: not significant

The tumor microenvironment was further analyzed to investigate the antitumor mechanism of PTX@CAR-Exos. CD8^+^ T cells play a key role in inhibiting the proliferation of tumor cells, and a high ratio of CD8^+^ T cells indicates remarkable bioactivity in eliminating tumors directly or indirectly through the release of granzyme B and perforin or the secretion of TNF-α and IFN-γ [47]. To establish whether PTX@CAR-Exos influenced the CD8^+^ T cell activation, the infiltration of CD8^+^ T cells was analyzed by flow cytometry (Fig. 6A, C). Results showed that CAR-Exos treatment moderately increased CD8^+^ T cells to 34.8% compared to the T-Exos treated group (31.9%). However, this difference was not significant. Importantly, a significant enhancement of the percentage of tumor-infiltrating CD8^+^ T cells (42.5%) was observed following PTX@CAR-Exos treatment. The activation of T cells was also explored by analyzing the marker granzyme B, which presented a similar trend to the CD8^+^ T infiltration results (Fig. 6B, D).

Subsequently, we analyzed cytokine levels via ELISA to further evaluate the condition of the tumor microenvironment. IFN-γ and TNF-α were assessed because they are typical cytokines involved in many antitumor responses [48]. In contrast to T-Exos, increased levels of cytokines were detected in the CAR-Exos treated group, but the difference was not significant (Fig. 6E, F). However, compared with the T-Exos and CAR-Exos treated groups, significantly elevated concentrations of both TNF-α and IFN-γ were measured in the PTX@CAR-Exos treated group. We have proven that CAR-Exos may target and kill tumor cells directly via producing granzyme B and perforin, resulting in the release of tumor-associated antigens and activation of the immune response. However, our results suggested that CAR-Exos had little effect on promoting immune cell growth and cytokine release. In contrast, PTX loading into CAR-Exos stimulated antitumor activity and promoted cytokine secretion in vivo. Therefore, PTX@CAR-Exos in this study exhibited the targeted delivery of PTX to the tumor site, which not only exerted the tumor-killing effect of CAR-Exos but also reshaped the inhibitory conditions of the TME by improving the bioavailability of PTX, thereby achieving a superior anti-tumor therapeutic effect.

Furthermore, cytokine levels in the serum were measured to confirm the safety of the inhaled therapy. The results showed that inhaled CAR-Exos caused little change in IL-2, IL-6, and IFN-γ levels in peripheral blood of mice, which were the main components of a cytokine storm [49]. In contrast, there was an apparent rise in the above-mentioned cytokines following intravenous administration of CAR-T cells (Additional file 1: Fig. S8), consistent with reports by Fu et al. [10]. These data illustrated the remarkable potential of inhaled CAR-Exos as a safe and controllable therapy.

## Discussion

Paclitaxel (PTX) therapy is the first-line strategy for clinical treatment of NSCLC, and it has shown some potency [50]. However, patients usually suffer from severe side effects after treatment, and most of them are caused by the clinical formulation and route of PTX injection. Intravenous administration often induces systemic toxicity because of the nonspecific delivery of free PTX in circulation [51]. For this reason, PTX should be sent to the tumor site directly. Our study showed that CAR-T derived exosomes can act as targeted carriers and deliver PTX to the local tumor origin to improve therapeutic efficacy, and this is attributed to the specific anti-MSLN scFv on the surface of exosomes to recognize and bind to MSLN, which was widely expressed in lung cancer. Recent evidence has shown that CAR-T-derived exosomes retained the targeted CAR and effector molecules from the parental cells, both of which are key to how CAR-T cell therapy kills tumors [10, 52]. We proved that CAR-Exos expressed the granzyme B and perforins internally, which resulted in the death of MLSN-expressing tumor cells.

However, increasing amounts of evidence indicate that most intravenous exosomes accumulate in the liver and only a minor proportion become distributed in the lung [53]. Inhalation therapy has been shown to be a feasible way to deliver drug-loaded exosomes to treat many pulmonary diseases. In this study, PTX-loaded CAR-Exos was administered via inhalation, and most of the CAR-Exos gathered in the lungs of orthotopic tumor-bearing mice, thus realizing the targeted delivery of chemotherapeutic drugs and enhancing the elimination of tumor cells in pulmonary area. PTX has been proven to regulate immune cells in the TME to limit tumor proliferation [54]. In addition, PTX treatment also stimulated the release of cytotoxic molecules and tumor suppressor cytokines to improve the killing effect [55]. In our study, we analyzed the percentages of immune cells and cytokines in TME, and the data showed that PTX@CAR-Exos promoted the percentage of CD8^+^ T cells and levels of TNF-α and IFN-γ in TME, which facilitated the bioavailability of the drug and reshaped the inhibitory conditions of the TME.

Our strategy of inhalable chemotherapeutic drug-loaded CAR-Exos has enormous potential in the field of lung cancer treatment. First, a variety of hydrophobic drugs could be considered for loading into CAR-Exos for delivery including immune checkpoint inhibitors, small molecule targeted drugs, and antibodies [56]. Second, various ligands or proteins could also be expressed on exosomes to reshape the inhibitory tumor microenvironment [57]. Hence, this natural biological carrier may broaden and simplify the application of CAR-T-based cancer therapy. Additionally, in contrast to the excessive inflammatory response of CAR-T cells [58], their derived exosomes may be administered quantitatively. Moreover, the modification of CAR-Exos may be applied in more complex and heterogeneous tumors. Following engineering, exosomes may change the inhibitory conditions in the TME through targeting inhibitory factors such as immunosuppressive cytokines and immune checkpoints [59]. These results suggest that loading chemotherapeutic drugs into CAR-Exos may not only overcome the current obstacles inherent in CAR-T cell therapy for solid tumors and solve the problem of CAR-T cell exhaustion, but also reduce clinical adverse reactions. Therefore, this may be a safer and more effective approach for patients with lung cancer.

## Conclusions

In conclusion, we designed a novel strategy for the targeted therapy of NSCLC through inhaled PTX loaded CAR-Exos. After inhalation, the main biodistribution of PTX@CAR-Exos was observed to be the lungs, and the exosomes expressing anti-MSLN scFv as a vehicle exhibited some ability to target tumor cells, resulting in an accumulation of the drug in the tumor and increasing its efficacy. Thus, this novel strategy may reduce the side effects of PTX in non-target organs and improve its antitumor efficiency.

## Supplementary Information

**Additional file 1: Figure S1.** The purity of different batches of CAR-Exos. Exosome purity level required for preclinical studies: 1 × 10^11^ particle/mg. **Figure S2.** Representative TEM image of PTX@CAR-Exo. Scale bar: 100 nm. Size distribution and zeta potential of CAR-Exo and PTX@CAR-Exo measured by NTA and DLS. Data are presented as the mean ± SD of three biological replicates. **Figure S3.** Determination of the standard curve for detecting PTX using a spectrophotometer. Mass of PTX = 0.77636 × OD value − 0.01551. **Figure S4.** Storage stability of CAR-Exo or PTX@CAR-Exo at − 80 °C. The average size of PTX@CAR-Exo did not change within 30 days at − 80 °C. Data are presented as the mean ± SD of three biological replicates. **Figure S5.** Representative histological images for H&E staining were obtained from the lung, liver, spleen, heart, and kidney of mice with different treatments post inhalation. Scale bars: 100 µm. Body weights of control mice and mice receiving different treatments. Serum ALT, ALP, and AST levels in mice from different treatments were used as indicators of liver injury. Serum CRE and BUN levels in mice from different treatments were used as indicators of kidney function. Data are presented as the mean ± SD of three biological replicates. PBS, T-Exo, CAR-Exo, PTX@CAR-Exo. **Figure S6.** The targeting of CAR-Exos to the orthotopic lung cancer in vivo. The accumulation of inhaled CAR-Exos labeled with DiR post administration were measured by IVIS system. **Figure S7.** Survival analysis of tumor-bearing mice after CAR-Exos inhalation. *P < 0.05, **P < 0.01, ***P < 0.001, ****P < 0.0001, ns: not significant. **Figure S8.** Cytokine concentrations in mouse peripheral blood following intravenous CAR-T cells administration or inhalation of CAR-Exos. Data are presented as the mean ± SD of three biological replicates. *P < 0.05, **P < 0.01, ***P < 0.001, ****P < 0.0001, ns: not significant.

## Abbreviations

NSCLC: Non-small cell lung cancer
PTX: Paclitaxel
CAR: Chimeric antigen receptor
MSLN: Mesothelin
LLC: Lewis lung cancer cell line
scFv: Single-chain variable fragment
PTX@CAR-Exos: PTX is loaded into CAR-Exos
GFP: Green fluorescent proteins
HEK293: Human embryonic kidney 293 cell line
LDH: Lactate dehydrogenase
TEM: Transmission electron microscopy
NTA: Nanoparticle tracking analyzer
DLS: Dynamic light scattering
PI: Propidium Iodide
PBS: Phosphate buffer saline
T-Exos: T cell derived exosomes
CAR-Exos: CAR-T cell derived exosomes
H&E: Hematoxylin
TNF-α: Tumor necrosis factor α
IFN-γ: Interferon γ
TME: Tumor microenvironment
IL: Interleukin
