# Supplementary material for: Inhalable CAR-T cell-derived exosomes as paclitaxel carriers for treating lung cancer
Source: J Transl Med. 2023 Jun 12;21:383. doi: 10.1186/s12967-023-04206-3 (PMC10262566; doi:10.1186/s12967-023-04206-3)
Supplement: Supplementary file 1 — Additional file 1: Figure S1. The purity of different batches of CAR-Exos. Exosome purity level required for preclinical studies: 1 × 10^11^ particle/mg. Figure S2. Representative TEM image of PTX@CAR-Exo. Scale bar: 100 nm. Size distribution and zeta potential of CAR-Exo and PTX@CAR-Exo measured by NTA and DLS. Data are presented as the mean ± SD of three biological replicates. Figure S3. Determination of the standard curve for detecting PTX using a spectrophotometer. Mass of PTX = 0.77636 × OD value − 0.01551. Figure S4. Storage stability of CAR-Exo or PTX@CAR-Exo at − 80 °C. The average size of PTX@CAR-Exo did not change within 30 days at − 80 °C. Data are presented as the mean ± SD of three biological replicates. Figure S5. Representative histological images for H&E staining were obtained from the lung, liver, spleen, heart, and kidney of mice with different treatments post inhalation. Scale bars: 100 µm. Body weights of control mice and mice receiving different treatments. Serum ALT, ALP, and AST levels in mice from different treatments were used as indicators of liver injury. Serum CRE and BUN levels in mice from different treatments were used as indicators of kidney function. Data are presented as the mean ± SD of three biological replicates. PBS, T-Exo, CAR-Exo, PTX@CAR-Exo. Figure S6. The targeting of CAR-Exos to the orthotopic lung cancer in vivo. The accumulation of inhaled CAR-Exos labeled with DiR post administration were measured by IVIS system. Figure S7. Survival analysis of tumor-bearing mice after CAR-Exos inhalation. *P < 0.05, **P < 0.01, ***P < 0.001, ****P < 0.0001, ns: not significant. Figure S8. Cytokine concentrations in mouse peripheral blood following intravenous CAR-T cells administration or inhalation of CAR-Exos. Data are presented as the mean ± SD of three biological replicates. *P < 0.05, **P < 0.01, ***P < 0.001, ****P < 0.0001, ns: not significant. [file 12967_2023_4206_MOESM1_ESM.docx]

**Additional file**

**Inhalable CAR-T Cell-Derived Exosomes as Paclitaxel Carriers for Treating Lung Cancer**

Wei Zheng‡^1^, Tianchuan Zhu‡^2^, Lantian Tang‡^1^, Zhijian Li^3^, Guanmin Jiang*^2^, and Xi Huang*^1,2^

^1^ Center for Infection and Immunity, Guangdong Provincial Key Laboratory of Biomedical Imaging, The Fifth Affiliated Hospital of Sun Yat-Sen University, Zhuhai, 519000 Guangdong China.

^2^ Department of Clinical laboratory, The Fifth Affiliated Hospital of Sun Yat-sen University, Zhuhai, 519000 Guangdong China.

^3^ Foshan Fourth People's Hospital, Foshan, 528200 Guangdong China.

*Corresponding authors: huangxi6@mail.sysu.edu.cn (Xi Huang); jianggm3@mail.sysu.edu.cn (Guanmin Jiang)


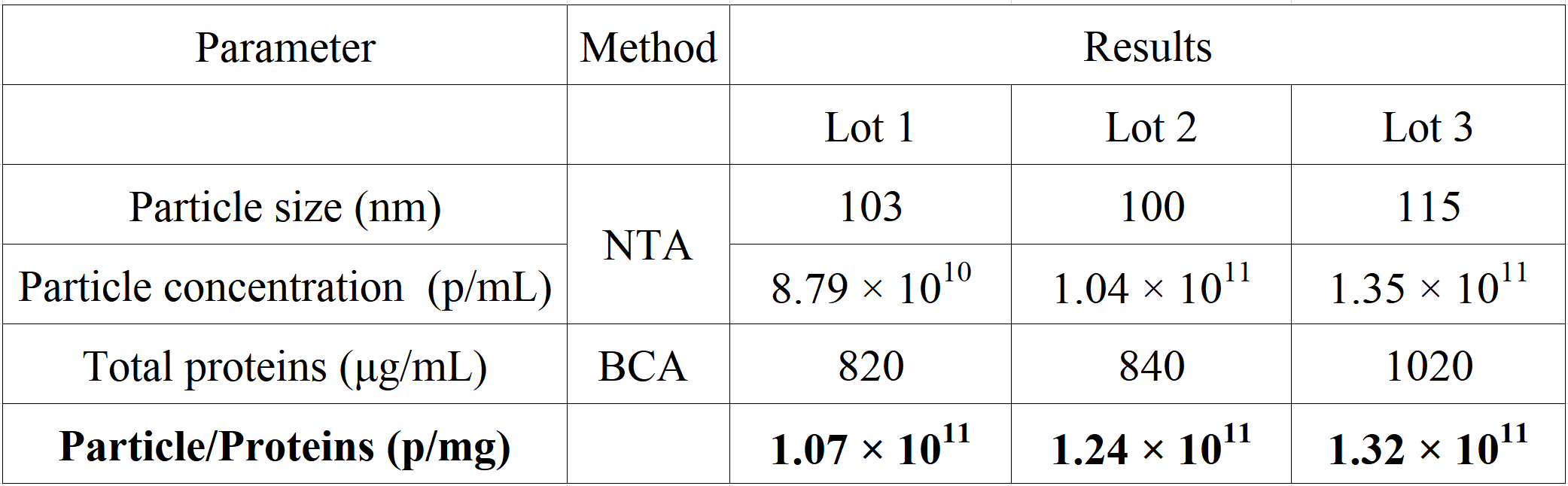


**Figure S1.** The purity of different batches of CAR-Exos (*n* = 3). Exosome purity level required for preclinical studies: 1 × 10^11^ particle/mg.


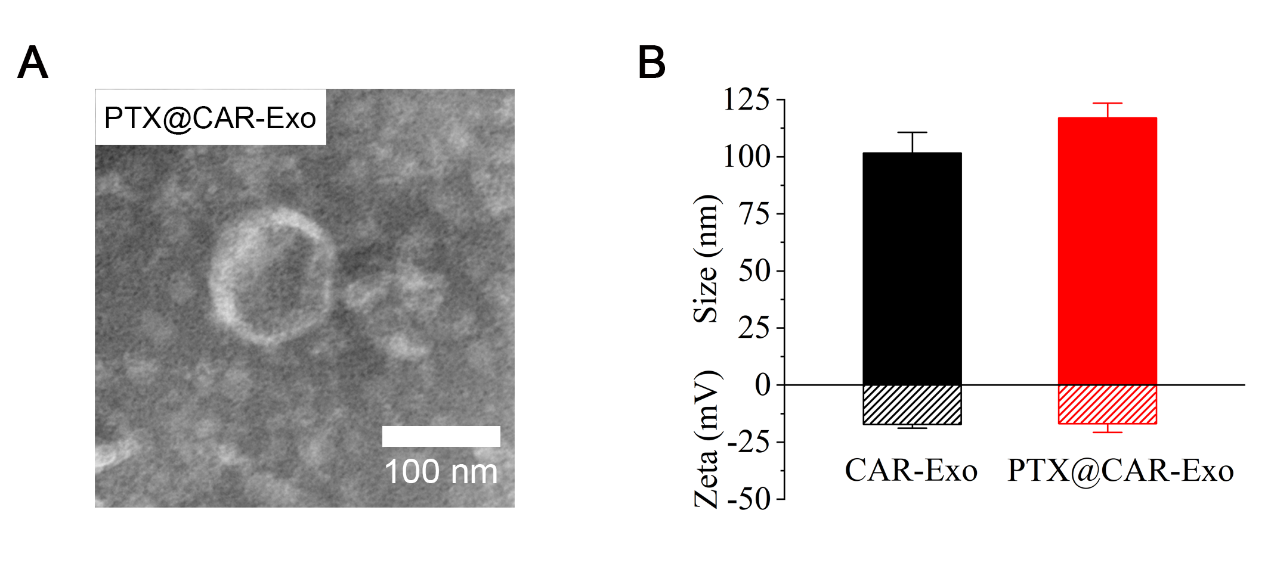


**Figure S2.** **(A)** Representative TEM image of PTX@CAR-Exo. Scale bar: 100 nm. **(B)** Size distribution and zeta potential of CAR-Exo and PTX@CAR-Exo measured by NTA and DLS. Data are presented as the mean ± SD of three biological replicates.


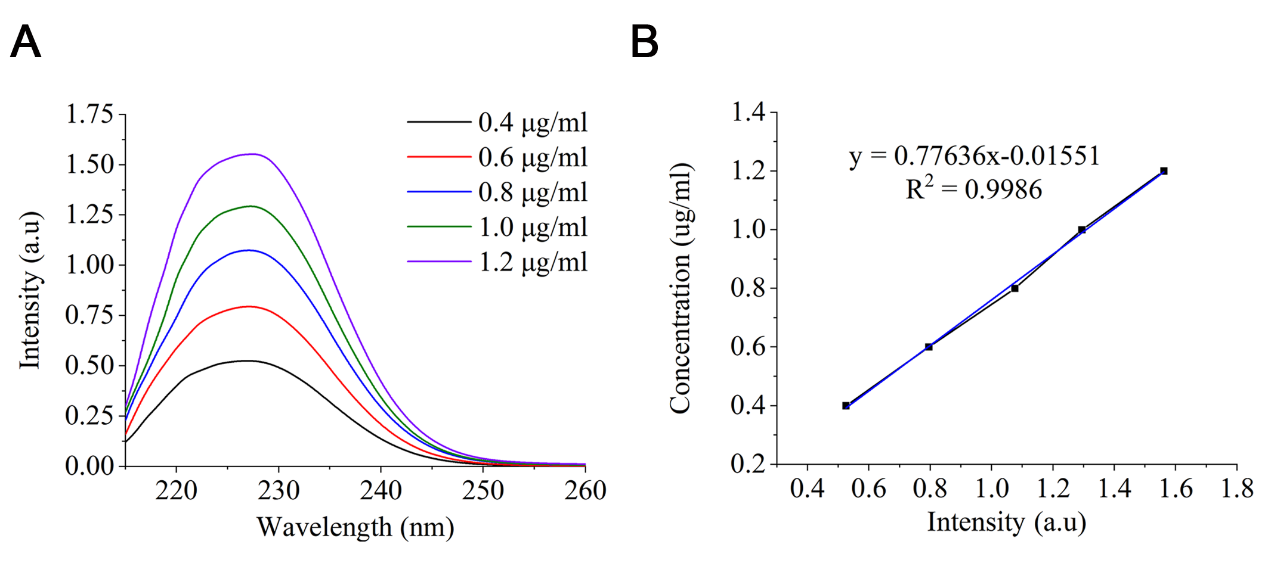


**Figure S3. (A)** Determination of the standard curve for detecting PTX using a spectrophotometer. **(B)** Mass of PTX (μg/mL) = 0.77636 × OD value - 0.01551.


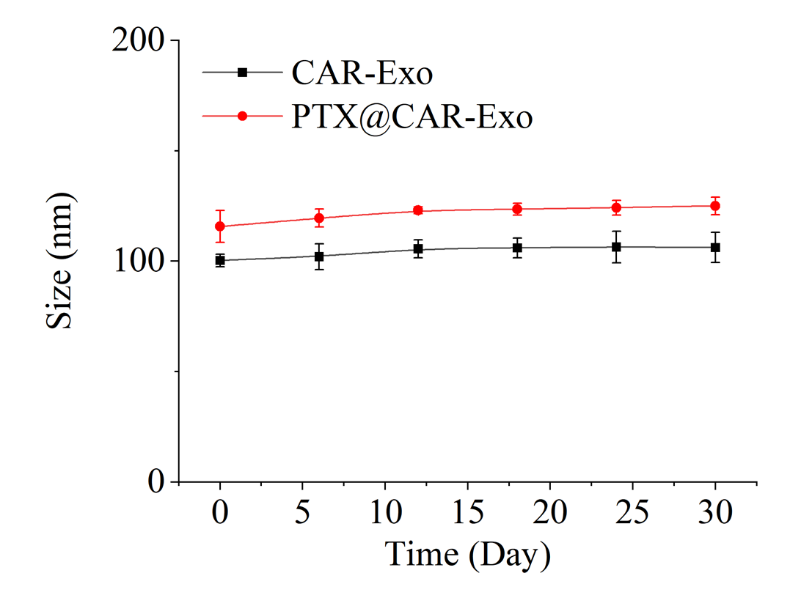


**Figure S4.** Storage stability of CAR-Exo or PTX@CAR-Exo at −80°C. The average size of PTX@CAR-Exo did not change within 30 days at −80°C. Data are presented as the mean ± SD of three biological replicates.


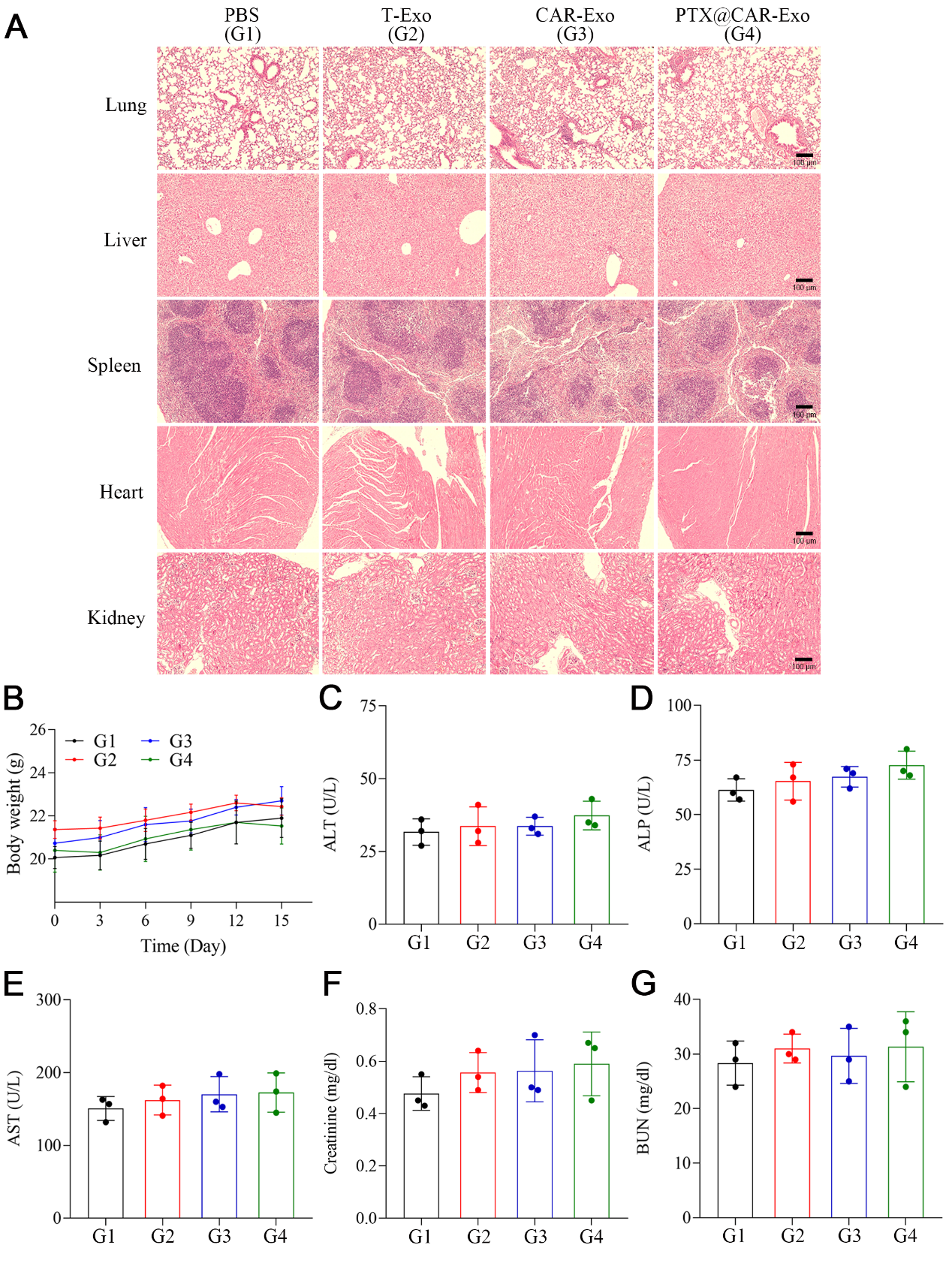


**Figure S5.** **(A)** Representative histological images for H&E staining were obtained from the lung, liver, spleen, heart, and kidney of mice with different treatments post inhalation. Scale bars: 100 µm. **(B)** Body weights of control mice and mice receiving different treatments. **(C–E)** Serum ALT, ALP, and AST levels in mice from different treatments were used as indicators of liver injury. **(F, G)** Serum CRE and BUN levels in mice from different treatments were used as indicators of kidney function. Data are presented as the mean ± SD of three biological replicates. PBS (G1), T-Exo (G2), CAR-Exo (G3), PTX@CAR-Exo (G4).


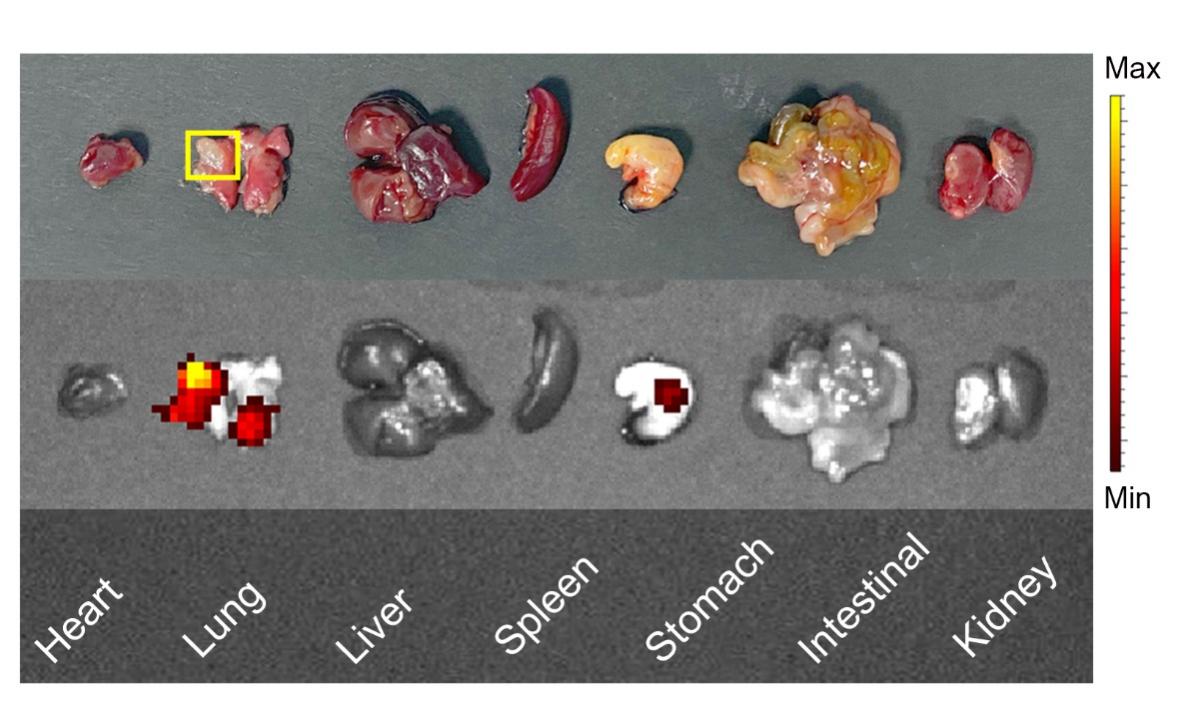


**Figure S6.** The targeting of CAR-Exos to the orthotopic lung cancer *in vivo*. The accumulation of inhaled CAR-Exos labeled with DiR post administration were measured by IVIS system.


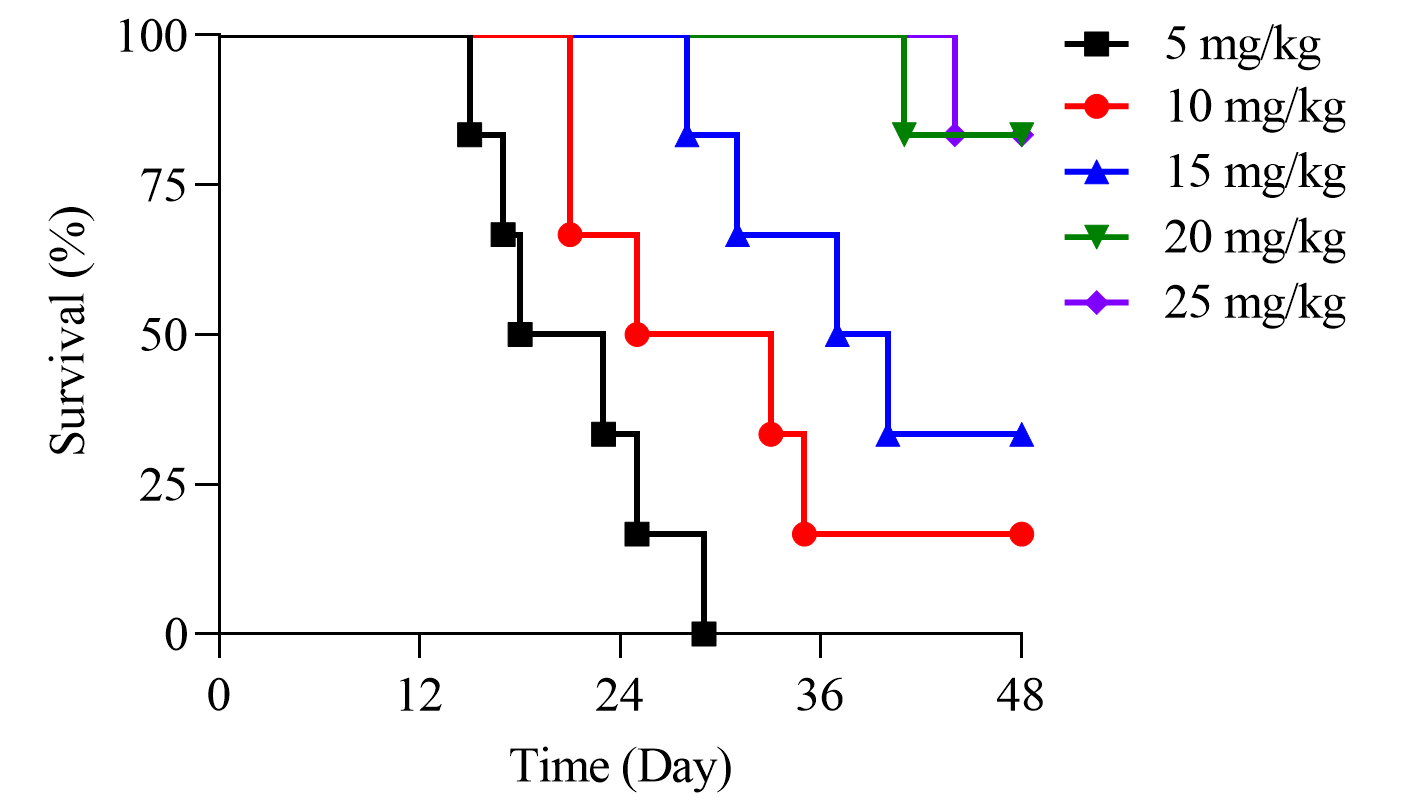


**Figure S7.** Survival analysis of tumor-bearing mice after CAR-Exos inhalation. **P* < 0.05, ***P* < 0.01, ****P* < 0.001, *****P* < 0.0001, ns: not significant.


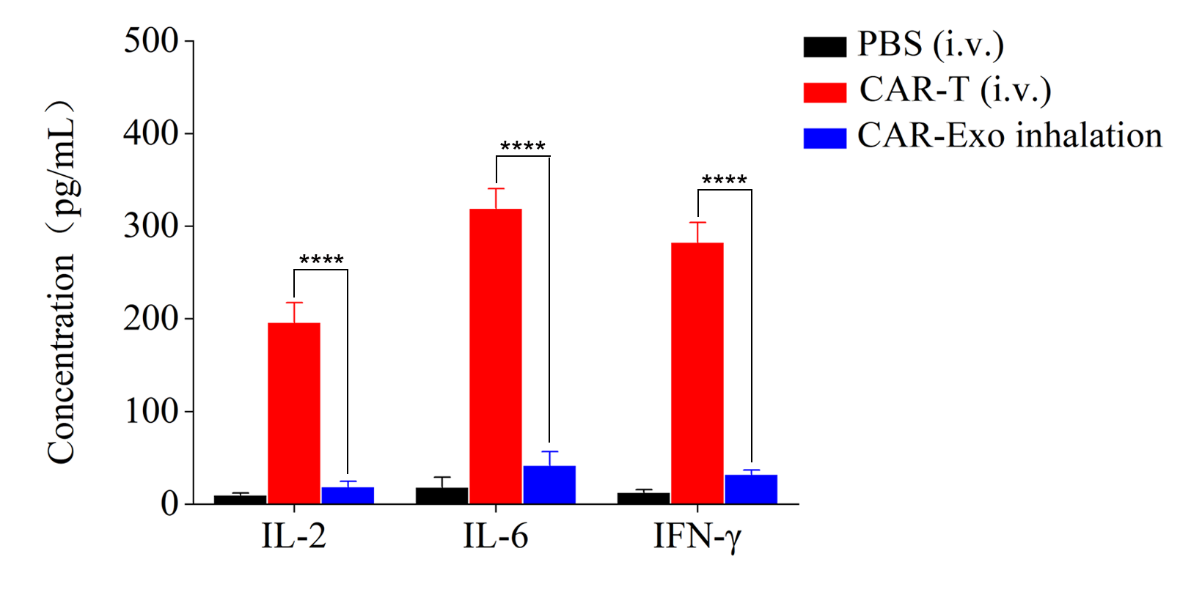


**Figure S8.** Cytokine concentrations (IL-2, IL-6, IFN-γ) in mouse peripheral blood following intravenous CAR-T cells administration or inhalation of CAR-Exos. Data are presented as the mean ± SD of three biological replicates. **P* < 0.05, ***P* < 0.01, ****P* < 0.001, *****P* < 0.0001, ns: not significant.
